# Assessment of microfilarial loads in the skin of onchocerciasis patients after treatment with different regimens of doxycycline plus ivermectin

**DOI:** 10.1186/1475-2883-5-1

**Published:** 2006-02-05

**Authors:** Alexander Yaw Debrah, Sabine Mand, Yeboah Marfo-Debrekyei, John Larbi, Ohene Adjei, Achim Hoerauf

**Affiliations:** 1 Institute for Medical Parasitology, University of Bonn, Sigmund-Freud-Str. 25, D-53105 Bonn, Germany; 2 Kumasi Centre for Collaborative Research in Tropical Medicine (KCCR), Kumasi, Ghana; 3 School of Medical Sciences, Kwame Nkrumah University of Science and Technology, Kumasi, Ghana; 4 Bernhard-Nocht Institute for Tropical Medicine, Hamburg, Germany

## Abstract

**Background:**

Infection with the filarial nematode *Onchocerca volvulus *can lead to severe dermatitis, visual impairment, and ultimately blindness. Since the currently used drug, ivermectin does not have macrofilaricidal or strong permanent sterilising effects on the adult worm, more effective drugs are needed to complement the use of ivermectin alone. *Wolbachia *endosymbiotic bacteria in filariae have emerged as a new target for treatment with antibiotics which can lead to long -term sterilization of the adult female filariae.

**Methods:**

In the Central Region of Ghana, 60 patients were recruited, allocated into four groups and treated with 200 mg doxycycline per day for 2 weeks, 4 weeks, 6 weeks respectively. Untreated patients served as controls. Some of the treated patients and the untreated controls were given 150 μg/kg ivermectin 8 months after the start of doxycycline treatment.

**Results:**

A follow up study 18 months post treatment showed that when using doxycycline alone there was a significant reduction of microfilarial (mf) loads in patients treated for either 4 or 6 weeks. However, there was no significant difference between the untreated controls and those given the 2 weeks regimen. Although no significant difference was demonstrated between the 4 and 6 weeks regimens, there was a trend observed, in that, microfilarial reduction appeared to have been greater following the 6 weeks regimen. Twelve months after ivermectin (i.e. 20 months after doxycycline) treatment, 8 out of 11 ivermectin-alone treated patients were mf-positive. In contrast, 1 out of the 7 patients treated for 4 weeks with doxycycline and none of the 4 patients treated for 6 weeks doxycycline (who were available for re-examination) were mf-positive after the combined treatment of doxycycline plus ivermectin treatment.

**Conclusion:**

Treatment of onchocerciasis with doxycycline for 4 weeks is effective. Nonetheless, mf reduction appeared to be greater in the 6 weeks regimen. It is recommended that until further studies are carried out i.e. 4 weeks treatment with doxycycline is proven equivalent to the 6 weeks, selected groups of onchocerciasis patients should be treated for 6 weeks with doxycycline. As discussed earlier, this treatment should be accompanied by two doses of ivermectin.

## Background

Onchocerciasis, commonly known as river blindness, is caused by the filarial nematode, *Onchocerca volvulus *[1]. It is an important cause of visual impairment and dermatitis, affecting about 18 million residents in Africa and Latin America [2]. The infection is known to be endemic in 37 countries [2]. In 1995, the World Health Organization Expert Committee on Onchocerciasis estimated that 123 million people were at risk of contracting the infection, and about 18 million were infected of whom 270, 000 were blind and 500, 000 severely visually impaired [3]. A recent study has shown a direct association between microfilarial load and excess mortality in onchocerciasis patients [4].

The world community aims to eliminate onchocerciasis as a public health problem [5]. Since the close down of the Onchocerciasis Control Programme (OCP) in West Africa at the end of 2002, all subsequent onchocerciasis control was transferred to the participating countries [6], and it is almost entirely based on periodic mass treatment with ivermectin using community directed treatment with ivermectin (CDTI) as in the African Programme for Onchocerciasis Control (APOC) [7]. APOC relies on community-based mass distribution of ivermectin once a year. It is accepted that APOC in its current form might not stop transmission completely [8,9]. The crucial problem is that ivermectin leads to depletion of skin microfilariae (mf) for only a few months, followed by reappearance of mf within one year at levels of more than 20% of that at pre-treatment [10-12] and this mf (microfilaria) density seems sufficient for transmission to continue.

In order to achieve elimination of onchocerciasis as a public health problem, ivermectin has to be applied annually for 10 - 20 years or more [3,5,13]. This is due to the longevity of adult worms, which can live for 9 - 14 years [14] and are not killed by ivermectin. Ivermectin given at shorter intervals of three- or six-months, rather, than the standard annual treatment may lead to excess mortality in the adult stages of *O. volvulus *[15-18]. However, more recent meta-analysis of extensive data from former OCP areas has shown that even semi-annual mass treatment of ivermectin for several years, followed by cessation e.g. due to political unrest, has resulted in high rates of recrudescence [6]. Ivermectin does not strongly affect early embryogenesis [19], with the results indicating that there is not a permanent sterilising effect. Indeed, microfilariae (mf) continue to be released and re-emerge in the skin of treated onchocerciasis patients several months after treatment. A review of the impact of 10 - 12 years of ivermectin treatment revealed that ivermectin was very effective in controlling the public health aspect of the disease. However, elimination of transmission proved difficult [6]. It is, therefore, unlikely that the scheme of ivermectin used currently can provide a complete solution to onchocerciasis. A recent conference on the eradicability of onchocerciasis concluded that eradication is not feasible with the present tools alone [13]. In addition, sub-optimal efficacy of ivermectin and/or ivermectin resistance in humans has been reported in onchocerciasis patients in Ghana where despite multiple treatments with ivermectin, microfilaridermia persisted in some patients [20,21]. It is important, therefore, to continue the development of alternative drugs for the treatment of onchocerciasis. Particularly useful would be a drug which kills or permanently sterilises the adult worms, thus ensuring a more definite impact of control on the reservoir of the parasite and possibly achieving finally its eradication [5]. There is, therefore, a pressing need for new antifilarial drugs which have macrofilaricidal efficacy, or which show total and long-lasting suppression of embryogenesis, in order to complement microfilaricides such as ivermectin [5].

Members of the tetracycline group of antibiotics have recently been proven to have antifilarial effects in animals [22,23] and in humans [24,25]. Their action is based on the targeting of *Wolbachia *endobacteria which exist in most filarial species [26].

A first study carried out on the effects of doxycycline treatment in onchocerciasis patients suggests that treatment with 100 mg per day for six weeks is effective in sterilising the female adult worms [25,27]. However, further investigations are needed to find out if shorter treatment regimens with a higher dose of 200 mg per day would be equally effective. Therefore, in the present study, we analysed the mf loads for up to 18 months after the beginning of doxycycline treatment in patients treated either with doxycycline alone using different treatment regimens, with ivermectin alone, or with doxycycline plus ivermectin.

## Methods

### Study site and patient recruitment

The study was carried out in 3 selected villages namely Akropong, Buabinso, and Denkyira Fosu, all in the Upper Denkyira District in the Central Region of Ghana, along the Offin river which is an endemic area for onchocerciasis [27]. It is a forest zone outside the Onchocerciasis Control Programme (OCP) area. The community prevalence of onchocerciasis in the study villages were 70%, 83% and 82% for Akropong, Buabinso, and Denkyira Fosu respectively (RD Horstmann et. al., unpublished data). The study was carried out from January 2000 to December 2002. Community mass anti-filarial treatment with ivermectin had started in March 1999 in the district by the Ministry of Health, but the district health administration could not give us the treatment coverage for the study villages.

The study design was approved by both the Ethical Committees of the School of Medical Sciences of the Kwame Nkrumah University of Science and Technology, Kumasi, and the Medical Board Hamburg, Germany. The study procedure and the symptoms of the potential side effects of doxycycline were explained to the participants. The patients were asked to report any side effects experienced in the course of the treatment period. Verbal consent was given by each participant. All those taking part in the study were informed that they could drop out of the study at any time they wanted.

### Study exclusion and inclusion criteria

Volunteer onchocerciasis patients aged 18–50 years were examined for palpable nodules and other chronic manifestations of onchocerciasis. Skin biopsies were taken from patients with nodules and their mf were counted, in order to enrol 87 patients for the study. Inclusion criteria were as follows: Volunteer onchocerciasis patients aged 18–50 years, who were in good general health conditions, and had a mf density of 5 mf or more per skin snip (Table 1). Exclusion criteria for the study were: abnormal hepatic and renal profiles (alkaline phosphatase >200 U/l, glutamate pyruvate transaminase >50 U/l, gamma glutamyl transpeptidase >28 U/l and creatinine >1.2 mg/100 μl) (measured by stick-chemistry) (Reflotron^®^, Roche Diagnotics, Mannheim, Germany), regular intake of other drugs, known mental illness, or other acute or chronic diseases. Women were not selected for doxycycline treatment but were included as control patients and offered ivermectin treatment and nodulectomy.

**Table 1:** Microfilariae/skin snip of patients selected for the study

Mf/skin snip	5–10	11–20	21–50	Above 50	Total
Number of patients	3	12	29	43	87

### Microfilarial count before therapy

Corneoscleral punches (Holth and modified Walser) were used to take bloodless skin biopsies in the study and this yielded skin snip weights of approximately 1.5 - 5.0 mg. Two skin biopsies, one from the upper part of each buttock were taken from each patient before treatment.

Each skin biopsy was immersed in 100 μl of 0.9% NaCl solution in a separate well of a 96-well round bottom microtitre plate (Nunc, Roskilde, Denmark) and labelled with the participant's code number. The wells of the plates were then covered with adhesive tape to prevent evaporation and spilling of the contents during transport to the laboratory at the district hospital in Dunkwa. The skin biopsies in the plates were incubated overnight at room temperature to allow the emergence of the mf into the saline solution. The solution in each well was thoroughly mixed and pipetted onto a glass slide for microscopic examination. Microfilariae were then counted using 63-fold magnification of a microscope. Each skin biopsy, after blotting to remove excess moisture, was weighed using a Sartorious electronic balance (Göttingen, Germany), and the number of mf from each biopsy expressed as the number of mf per milligram (mf/mg) of skin. The geometric mean of the mf from the two skin biopsies from each patient was calculated and used as a measure of intensity of infection.

### Doxycycline treatment

**Figure 1:**
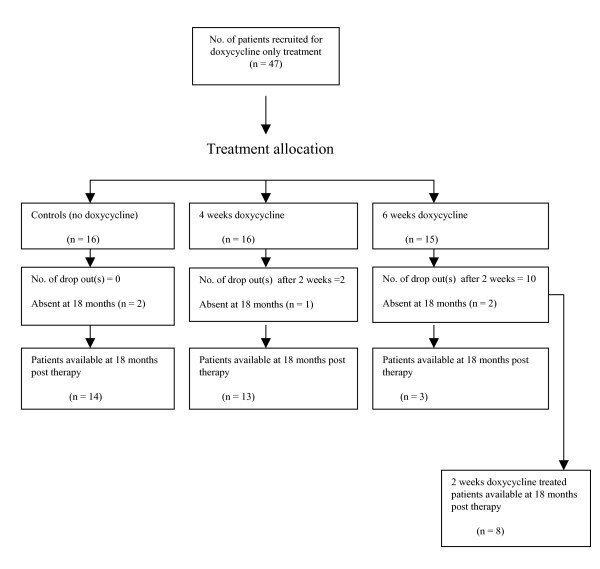


In all, 47 patients took part in the doxycycline-only treated study. These patients were allocated to four groups (Fig. 1) as indicated below and treated with doxycycline (Vibramycin^®^, Pfizer) at a dose of 200 mg per day under the direct observation of the team clinician who also monitored participants for adverse reactions. Group 1: 12 patients received doxycycline for 2 weeks; group 2: 14 patients received doxycycline for 4 weeks; group 3: 5 patients received doxycycline for 6 weeks; and Group 4: 16 patients served as controls and received no doxycycline treatment during the study period. Patients of group 1 had been assigned to either group 2 or 3 but could either not complete the full course or voluntarily dropped out of the doxycycline administration. For instance, 10 patients in group 1 had been assigned to group 3 (Fig. 1) but withdrew after 2 weeks because they had to travel to their hometown in another region for an emergency event, whereas two patients assigned to group 2 voluntarily withdrew from the doxycycline administration. They were all followed-up during the study period.

### Ivermectin treatment

**Figure 2:**
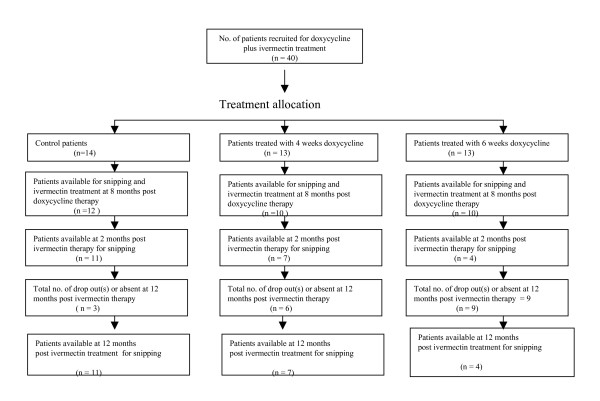


Forty patients (Fig. 2) comprising of 14 untreated controls, 13 patients from the 4 weeks doxycycline treated regimen and 13 from the 6 weeks doxycycline treated regimen were offered ivermectin 8 months after the start of doxycycline treatment. Each patient received a single dose of 150 μg/kg of ivermectin after snipping. Adverse reactions to ivermectin were noted using a medical questionnaire by a clinician on the team.

### Microfilarial count after therapy

Two skin biopsies, one from the upper part of each buttock were taken from doxycycline-only treated patients at 18 months after the start of doxycycline treatment, while two skin biopsies were taken at 2 and 12 months after the ivermectin administration from patients treated with ivermectin as has already been described above. Since ivermectin was administered 8 months after doxycycline treatment, these two time points (2 and 12 months) corresponded to 10 and 20 months respectively after doxycycline treatment.

### Statistical analysis

Data were summarised as geometric means (GM). Microfilarial geometric mean intensities were calculated as antilog {(∑log(x+1))/n}-1, with x being the mean of mf/mg of skin and n the number of individuals examined [25].

The efficacy of the various treatment regimens was assessed comparing the proportion of mf-positive individuals before and after treatment using Fisher's exact test. Differences in GM of mf before and after treatment among the various treatment regimens were analysed using ANOVA multiple comparison Post Hoc test Bonferroni, and differences within the same treatment groups were assessed using Wilcoxon Signed Rank test. A result of p < 0.05 was considered significant. Independence of data was tested using multivariate analysis (StatView^®^).

## Results

### Participation of patients at follow-up examinations

In all, 47 patients were recruited for the doxycycline-only treated study but nine patients dropped out of the study leaving 38 (Fig. 1). Of these nine patients, one died apparently of strangulated inguinal hernia, another died of HIV and seven individuals could not be re-examined because they had moved from their villages. Forty patients were also recruited for the doxycycline plus ivermectin study. Eighteen dropped out or were absent at 12 months post ivermectin therapy leaving 22 (Fig. 2).

### Pre-treatment findings

The major treatment groups, the doxycycline-only-treated, ivermectin-only-treated, doxycycline plus ivermectin-treated, and untreated controls were similar in age, skin mf count, body weight and distribution of clinical features. The most common skin lesions were atrophy of the skin involving the lower half of the body and a spotty depigmentation known as 'leopard skin'. Female patients in the study had higher mf loads expressed as a geometric mean (n = 15, GM = 17.1) than the males (n = 72, GM = 11.5). This difference was not significant using the students t-test (P = 0.4093, Student's t-test).

### Adverse reactions during and after treatment

The different treatment regimens with doxycycline were all well tolerated in the study population with few and only mild adverse reactions. Two patients experienced transient diarrhoea and another person had diarrhoea with bloody stool(s) for two days. No serious side effects from doxycycline treatment were documented during the treatment period. Adverse reactions such as itching, pruritus, rash, fever, swellings, swollen lymph nodes, and headache most directly associated with microfilarial killing occurred only after ivermectin administration. Symptoms of adverse reactions were noted using a medical questionnaire by a clinician on the team.

### Microfilarial counts recorded for doxycycline only treatment

Skin mf loads declined after treatment in all the treatment regimens including the control patients (Table 2). However, very low mf counts were recorded as less than 1 mf/mg for the 4 and 6 weeks groups compared to higher mf loads in the 2 weeks and control groups 18 months after treatment (Table 2).

**Table 2:** Geometric means (GM) of mf/mg skin of patients treated with doxycycline only (For significance, see Tables 3 and 4)

	Treatment group			
	
	Control	2 weeks	4 weeks	6 weeks
Mf-positives/persons examined	14/14	8/8	13/13	3/3
% of mf-positive patients	100 %	100 %	100 %	100 %
GM before treatment	16.7	12.2	10.8	14.1
Percentage of GM	100 %	100 %	100 %	100 %
				
Mf-positives/persons examined	14/14	7/8	7/13	1/3
% of mf-positive patients	100 %	87.5 %	53.8 %	33.3 %
GM at 18 months *	4.1	4.3	0.7	0.1
% of pre-treatment GM	24.6 %	35.2 %	6.5 %	0.7 %

* = Months after the start of doxycycline treatment.

Table 3 summarises the results of the relevant significance tests comparing mf densities of the various treatment regimens. In addition, the comparison of these observed differences in the proportions of mf-positive people yielded the following important results. Significant differences were observed between controls and the 4 and 6 weeks regimens (Table 4) but there were no significant differences between controls and 2 weeks regimen. This indicates that treatment with doxycycline for 4 or 6 weeks could be effective in treating onchocerciasis.

**Table 3:** Comparison of significant differences* of skin GM mf of the various treatment regimens 18 months after therapy.

Treatment regimen	P-value before treatment	P-value 18 months after treatment
Controls vs. 2 weeks	0.1865	0.2262
Controls vs. 4 weeks	0.4146	0.0042
Controls vs. 6 weeks	0.6286	0.0138
2 weeks vs. 4 weeks	0.0577	0.0012
2 weeks vs. 6 weeks	0.1419	0.0028
4 weeks vs. 6 weeks	0.8933	0.5628

* = ANOVA Post Hoc (Bonferroni/Dunn) test

**Table 4:** Comparison of significant differences* from mf positive/patients examined following the various treatment regimens 18 months post treatment.

Treatment regimen	P-value before treatment	P-value 18 months after treatment
Controls vs. 2 weeks	> 0.9999	0.3636
Controls vs. 4 weeks	> 0.9999	0.0058†
Controls vs. 6 weeks	> 0.9999	0.0221†
2 weeks vs. 4 weeks	> 0.9999	0.1736
2 weeks vs. 6 weeks	> 0.9999	0.1515
4 weeks vs. 6 weeks	> 0.9999	> 0.9999

* = Fisher's exact test

† = Significant difference

### Microfilarial counts recorded for doxycycline plus ivermectin treatment

Ivermectin was administered to both doxycycline-treated and untreated control patients 8 months after the start of doxycycline treatment. The skin mf counts fell to zero 2 months after the administration of ivermectin in both doxycycline-treated and untreated control patients (Tables 5). However, mf re-appeared in 8 out of 11 ivermectin-only-treated patients within 12 months (Table 5) after the ivermectin treatment. In contrast, the seven patients treated with doxycycline for 4 weeks before ivermectin administration remained mf-negative with the exception of one patient who had a very few mf at 12 months after treatment (Table 5). All the four patients treated for 6 weeks with doxycycline, however, remained mf-negative with none of the patients having mf in the skin (Table 5). This suggests that 4–6 weeks doxycycline plus ivermectin treatment is clearly more effective in reducing and maintaining low microfilaridermia for 12 months than the use of ivermectin alone. There was a significant difference between the ivermectin only treated regimen and the doxycycline plus ivermectin regimens (Table 6), but no difference between the 4 weeks doxycycline plus ivermectin and the 6 weeks doxycycline plus ivermectin regimens when ivermectin was administered 8 months after doxycycline treatment (Table 6).

**Table 5:** Geometric means (GM) of mf/mg skin of patients treated with doxycycline plus ivermectin*

	Treatment group		
	
	Ivermectin only	4 weeks doxycycline + ivermectin	6 weeks doxycycline + ivermectin
Mf-positives/persons examined	11/11	7/7	4/4
% of mf-positive patients	100 %	100 %	100 %
GM before doxycycline treatment	11.4	7.6	11.8
% of pre-treatment GM	100 %	100 %	100 %
			
Mf-positives/persons examined	11/11	7/7	4/4
% of mf-positive patients	100 %	100 %	100 %
GM* when ivermectin was administered	6.2	3.8	2.5
% of pre-treatment GM	54.4 %	50.0 %	21.2 %
			
Mf-positives/persons examined	0/11	0/7	0/4
% of mf-positive patients	0 %	0 %	0 %
GM at 2 months **	0	0	0
% of pre-treatment GM	0 %	0 %	0 %
			
Mf-positives/persons examined	8/11	1/7	0/4
% of mf-positive patients	72.7 %	14.3 %	0 %
GM at 12 months **	1.2	0.02	0
% of pre-treatment GM	10.8 %	0.3 %	0 %

* = Ivermectin was administered 8 months after doxycycline treatment. ** = Months after ivermectin treatment.

**Table 6:** Comparison of significant differences between the treatment regimens 12 months post ivermectin (20 months after doxycycline) treatment.

Treatment regimen	Geometric mean*		Mf positive/persons examined^‡^	
	
	P value before treatment	P value after treatment	P value before treatment	P value after treatment
Controls vs. 4 weeks	0.8265	0.0056	>0.9999	0.0498
Controls vs. 6 weeks	0.6138	0.0095	>0.9999	0.0256
4 weeks vs. 6 weeks	0.7623	0.2337	>0.9999	>0.9999

* = ANOVA Post Hoc (Bonferroni/Dunn) test

^‡ ^= Fisher's exact test

## Discussion

There is an urgent need for a long-term sterilising or macrofilaricidal drug effective against *O. volvulus *to complement ivermectin. In addition, new drugs with modes of action different to ivermectin must urgently be developed so that they are available should ivermectin resistance develop [6], a scenario that may not be too far in the future [20,21]. Treatment with 6 weeks doxycycline is effective. While this duration of treatment is considered too long for mass treatment [28], shorter regimens are also preferable for individual treatment and to facilitate better compliance.

The purpose of this study was to compare different regimens of doxycycline treatment so that we could determine the minimum effective duration necessary for the control of onchocerciasis.

Treatment with doxycycline for 2 weeks was not effective for onchocerciasis control as there was no significant difference between the untreated control groups and the 2 weeks doxycycline-treated groups 18 months post therapy (Tables 3 and 4). Treatment with doxycycline for either 4 or 6 weeks on the other hand, had an effect at the same time point (Tables 3 and 4), which suggests that both the 4 and the 6 week treatment regimens might be equally effective for onchocerciasis treatment.

One patient treated for 4 weeks with doxycycline plus ivermectin a had low mf count (GM = 0.02) (Table 5) in the skin 12 months after ivermectin treatment, whereas none of the patients treated for 6 weeks (plus ivermectin) had any mf. The low numbers of mf left in the skin may be an indication that either the 4 weeks regimen was not as beneficial as the 6 weeks regimen, or it could be due to the relatively larger sample size of the 4 weeks group (n = 7) compared to the smaller sample size of the 6 weeks group (n = 4). This, therefore, calls for a study with a larger sample size (which is underway) to compare the efficacy of the 4 and 6 weeks regimens. Nonetheless, what is clear from this study is that a 2 weeks regimen is not effective for the treatment of onchocerciasis. Four weeks is effective but may not be as beneficial as the 6 weeks regimen.

The observed reduction of mf in the control groups and the 2 weeks doxycycline treated patients cannot be fully explained. However, natural fluctuation unrelated to drug treatment cannot be ruled out as observed in both veterinary [29] and human studies [30-32]. When the degree of reduction of mf loads within the same treatment groups were compared 18 months post therapy using the Wilcoxon Signed Rank test, there was no significant difference between the drop of mf loads in the control patients (P= 0.0648) and those observed in the 2 weeks treatment group (P= 0.1282). In contrast there was a significant difference in the 4 weeks treatment group (P = 0.0039). Ironically, even though there was more than a 99% reduction of mf in the 6 weeks treatment group (Table 2), there was no significant difference when a paired test was performed using the Wilcoxon Rank test (P= 0.1088). This lack of impact in the 6 weeks regimen was due to the small sample size of the group. The significant differences between the control and the 4 and 6 weeks regimens (Tables 3 and 4) coupled with the clear significant differences between ivermectin-only treated and 4–6 weeks doxycycline plus ivermectin groups showed that both the 4 and 6 weeks doxycycline treatment regimens show a significantly improved embryostatic effect as compared with the use of ivermectin alone.

The profound and significant reduction of mf loads produced by the combined treatment of doxycycline and ivermectin is consistent with earlier reports [24,25,27] which used 100 mg doxycycline per day. This clearly shows that 4–6 weeks treatment with 200 mg doxycycline per day of patients with moderate to high intensities of *O. volvulus *infection is well tolerated and that 18 months post therapy, patients treated for 4–6 weeks had very low mf. Combined treatment of 4 or 6 weeks doxycycline plus ivermectin both suppressed microfilaridermia in onchocerciasis patients up to 12 months post therapy. This suggests an embryostatic effect for doxycycline [27]. This study has shown, that a 4 weeks regimen was effective, but might not be as beneficial as the 6 weeks regimen. It is, therefore, recommended that until a clear benefit or otherwise of a 4 weeks treatment regimen in a larger study is ascertained, the use of 6 weeks doxycycline treatment should be further explored for special situations in onchocerciasis control. As reported earlier [33-35], a single controlled application of doxycycline for 6 weeks accompanied by two additional single doses of ivermectin in onchocerciasis patients might prove more cost-effective than annual ivermectin treatment alone in special situations. One dose should be administered during doxycycline treatment and the other approximately 4–6 months later to eliminate mf developing in the early weeks of doxycycline treatment. This could reduce the transmission to a low level if a substantial part of the population in a particular focus is covered. This combined treatment should be explored for onchocerciasis control in special conditions, especially as curative treatment for individuals leaving onchocerciasis endemic areas permanently and wanting to remain free of mf in the long-term, or in areas where there is apparent existence of sub-optimal efficacy of ivermectin as reported in some onchocerciasis-endemic foci in Ghana recently [20,21].

## Conclusion

The purpose of this study was to find a shorter possible regimen that is effective for onchocerciasis treatment with doxycycline. Treatment for only 2 weeks with 200 mg doxycycline per day was not effective at stoppping microfilariae production. In contrast, both treatments for either 4 weeks or for 6 weeks with 200 mg per day were effective. The results of this study suggest that the 4 weeks regimen has the potential to be used for the treatment of onchocerciasis. There was no significant difference between the 4 and 6 weeks regimens, but the reduction in microfilarial numbers appeared to have been greater after 6 weeks. It is, therefore, recommended that further studies focus on the 4 weeks regimen, in a larger and preferably, in a double blind study. Presently, 6 weeks doxycycline treatment can be recommended for special situations. Since previous studies have shown that 6 weeks with 100 mg doxycycline per day are effective at stopping microfilariae production, this dose may be sufficient.

## Competing interests

The author(s) declare that they have no competing interests.

## Authors' contributions

AYD participated in patient recruitment and doxycycline daily treatment, performed the microfilaridermia analysis, compiled the data, and drafted the manuscript.

SM participated in patient recruitment, performed the blood taking, and doxycycline daily treatment.

YM participated in the negotiations with the village elders, patient recruitment and doxycycline daily treatment.

JL participated in patient recruitment and doxycycline daily treatment.

OA did the preparatory studies for the selection of villages, negotiated with the District Health Management and the village elders, and applied for the ethical clearance.

AH conceived the idea, designed and supervised the study, and edited the final manuscript version.

All authors read and approved the final manuscript.
